# The Exact Distributions of *F*_IS_ under Partial Asexuality in Small Finite Populations with Mutation

**DOI:** 10.1371/journal.pone.0085228

**Published:** 2014-01-21

**Authors:** Solenn Stoeckel, Jean-Pierre Masson

**Affiliations:** INRA, UMR1349 Institute for Genetics, Environment and Plant Protection, Le Rheu, France; University of Idaho, United States of America

## Abstract

Reproductive systems like partial asexuality participate to shape the evolution of genetic diversity within populations, which is often quantified by the inbreeding coefficient *F*_IS_. Understanding how those mating systems impact the possible distributions of *F*_IS_ values in theoretical populations helps to unravel forces shaping the evolution of real populations. We proposed a population genetics model based on genotypic states in a finite population with mutation. For populations with less than 400 individuals, we assessed the impact of the rates of asexuality on the full exact distributions of *F*_IS_, the probabilities of positive and negative *F*_IS_, the probabilities of fixation and the probabilities to observe changes in the sign of *F*_IS_ over one generation. After an infinite number of generations, we distinguished three main patterns of effects of the rates of asexuality on genetic diversity that also varied according to the interactions of mutation and genetic drift. Even rare asexual events in mainly sexual populations impacted the balance between negative and positive *F*_IS_ and the occurrence of extreme values. It also drastically modified the probability to change the sign of *F*_IS_ value at one locus over one generation. When mutation prevailed over genetic drift, increasing rates of asexuality continuously increased the variance of *F*_IS_ that reached its highest value in fully asexual populations. In consequence, even ancient asexual populations showed the entire *F*_IS_ spectrum, including strong positive *F*_IS_. The prevalence of heterozygous loci only occurred in full asexual populations when genetic drift dominated.

## Introduction

Reproductive systems define how genetic diversity is transmitted through generations thus they highly constrain the evolution of species. Many species relevant to human activities and ecosystems are partially asexual, meaning that they can reproduce both through sexual and asexual (equally named clonal) events [1]–[3]. Theoretical population genetics of partially asexual species has received little attention so far [4] and there is an ongoing debate on the effects of asexuality on genetic diversity and how such effects can be used to identify asexual species [5]. Indeed, theoretical studies about the genetic consequences of partial and full asexuality have only focused on the mean expected values of some population genetics parameters. In consequence, to disentangle evolutionary forces acting on such populations, applied studies may only compare their multiple quantitative measures of genetic diversity to theoretical average tendencies [6]–[8] and a large amount of the quantitative information contained within molecular data is wasted for evolutionary interpretations. Applied population genetics studies of partially asexual species lack of full frames of reference to compare their measured distributions of genetic parameters. Moreover, while we know that reproductive systems may have deep implications for the ecology and the evolution of species [9], we cannot rationally assess the evolutionary interests of sex, including genetic segregation, gamete fusion and massive recombination without understanding the full genetic consequences of partial and full asexuality [2], [10]–[14].

The population inbreeding coefficient, *F*_IS_ [15], is a classical measure of genetic diversity for polyploid organisms that allows biologists to assess the evolutionary processes acting on a population. *F*_IS_ is known to vary markedly according to mating systems, especially with the relative importance of sexuality and asexuality within populations [16], [17]. It constitutes the lowest level of *F*-statistics [15] and stands for the excess or deficit of heterozygotes occurring in a population as compared to Hardy-Weinberg proportions. Asexual events are expected to maintain heterozygosity within the offspring because, by limiting the segregation of alleles, it conserves the ancestral heterozygosity through generations. Moreover, asexuality is even expected to increase heterozygosity and decrease the probability of allele identity since alleles of the same gene may independently accumulate mutations over generations. However, this process known as the “Meselson effect” was formulated considering only one genome rather than a population of genomes [18]. Extending the same argument to many individuals shows that the mean heterozygosity of asexual populations should also increase [19], [20]. Empirical and theoretical results suggest that the effective rates of asexuality, the effective frequency of asexual events involved in producing the next generation in a population, is a key feature to understand the genetic evolution of those species [16]. The rate of asexuality is denoted *c* [19]. It ranges from 0, when populations reproduce only sexually, to 1 when populations reproduce only asexually. This rate is identical to *A* in [21], and shares common notion with *δ* [22] and with the length of the asexual seasons, *c* [23] but may have different impacts and implications.

The insights gained by mathematical and simulation modeling [19], [20], [22]–[26] raise unsolved questions, mainly because the theoretical expectations were previously formalized only for the first moment (mean) of the possible distributions of *F*_IS_. Intermediate asexual populations, defined as populations producing 0 to 90 percent of their descents using asexuality, are expected to exhibit similar mean *F*_IS_ values and variance to those obtained from fully sexual populations [19], [24]. This implies that intermediate rates of asexuality should have no effects on the average level of genetic diversity expected within populations. If true on the full range of genetic diversity, a strategy with a low frequency of sex (*c* around 0.9) would be optimal, considering that it would combine the potential genetic benefits of mixis in terms of heritability of genetic diversity and would reduce the costs of sex [27]. Conversely, to explain why we can still observe populations that steadily reproduce through partial asexuality, we may suppose that the balance between the genetic benefits of mixis and the costs of sex should vary with different rates of asexuality [12]. Empirical and field studies observe that partially asexual species show lower negative *F*_IS_ values at most loci when other direct evidences of asexuality argue for intermediate rates rather than full sexual species [17], [28]. This feature is thus commonly used to pragmatically detect asexual events in populations assumed to be sexual [16].

Sparsely studied, the second moments (variance) of the possible distributions of *F*_IS_ were only attempted using simulations [19], [26]. Highly asexual populations (0.9<*c*<1.0) should exhibit the topmost variance of *F*_IS_, distinctively lower than the ones expected in fully sexual and intermediate asexual populations (0.0<*c*<0.9), and slightly higher than those expected in fully asexual populations (*c* = 1) [19]. This topmost variance of *F*_IS_ expected in highly asexual populations was used to propose a way to qualitatively infer the rare occurrence of sexual events in such populations [29]. Yet, some field studies show shifting conclusions depending on whether they argue from *F*_IS_ variance or from other population parameters [8], [28], [30] indicating that this method may be not universal. Up to now, field studies still need to resort to expertise to identify, predict and infer the evolutionary forces ongoing on partially asexual species [4], [31], mostly because theoretical predictions only exist as mean expected values for some specific drift and mutation cases, rather than as full distributions of values computed for an extensive quantitative range of evolutionary forces.

As a better frame of reference to disentangle which evolutionary forces are affecting partially asexual species, we propose a raw population genetics model that provides the full exact distributions of *F*_IS_ ensued from an evolution with mutation in a finite population after an infinite number of generations. To achieve the mathematical description of those probabilities avoiding any continuous or “allelic pool” approximations for mathematical convenience [32], we derived the exact probabilities of all possible distributions of genotypes among the constitutive individuals of a finite population size as function of the rates of asexuality. By this approach, we were able to investigate the consequences of partial asexuality on discrete distributions of allelic identities and *F*_IS_, obtained from a single biallelic locus in a finite population with a steady reciprocal mutation flux between the two alleles. We also assessed how population sizes and mutation rates in interaction with the rate of asexuality impacted the probabilities to observe negative and positive *F*_IS_ values within populations and the full distribution of *F*_IS_. For better comparability, we also calculated the two traditional first moments (mean and variance) and expanded our analysis to the third (skewness) and the fourth (kurtosis) moments to unravel the kind of information that can be missed using only the two first moments to sum up the impacts of partial asexuality on the possible distributions of *F*_IS_. We hope that our predictions will help future biological studies to formalize hypotheses about the evolutionary forces, the biological traits and the ecological processes acting on populations, and to better assess the rates of asexuality estimated from genetic markers. This theoretical study constitutes a first step to take up the challenges expressed in recent perspectives to disentangle classic evolutionary forces from the consequences of such ‘odd’ mating systems [4], [33].

## Methods

### Our conceptual model

We aimed to build the simplest conceptual model with the objective to provide a comprehensive view of the genetic consequences of partial asexuality on a biallelic marker. We thus focused on the temporal change in genotypic frequencies at a diploid locus with two allelic states, *A* and *a*, obtainable from a reciprocal mutation rate *μ*, within a partially asexual finite population. Our model was based on genotypic frequencies rather than pools of alleles as recommended [32]. All change in the system was due to the mating system, genetic drift and mutation forces acting consistently through time expressed in generations. We further assumed no selection and no migration, even if mutation rate can be also interpreted as including some migration from external populations. Through asexual events, genetic drift acted at the genotypic level while, through sexual events, it acted at the allelic level. Population reproduced using discrete and non-overlapping generations. Within the population, the *N* individuals reproduced using asexuality at a rate *c*, called the rate of asexuality [19], [23], [26]. The genetic material was thus transmitted asexually at a rate *c* and sexually at a rate 1−*c*. Sexual reproduction followed the traditional random union of gametes implying pangamy and panmixy. In such sexual system, self-fertilization occurred at an average rate of 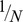 and alleles mutated during their transmission to the next generation. When an offspring resulted from an asexual event, he received the whole diploid genotype of its parent excepting mutations that occurred at similar rates during asexual and sexual reproduction.

### Mathematical development

We formalized a genotypic state 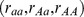 as a distribution of the *N* individuals of a population on the three possible genotypes *aa*, *aA*, *AA* that can be found at one biallelic locus. All the genotypic states were constrained by 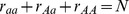. Thus a population of *N* individuals defined 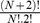 unique distributions of its *N* individuals within the three possible genotypic states. The genotypic frequencies at a generation *n* are thus 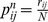 where *i* and *j* can be alleles *A* or *a*. The population size *N*, the mutation rate *μ* and the rate of asexuality *c* are supposed to be fixed all along the evolution of a population. We thus wrote the different transition probabilities from one generation to another.

The genotypic frequencies under asexual reproduction 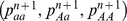 at the generation 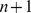 were expressed as a function of the mutation rate *μ* and the previous genotypic frequencies 
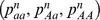
.
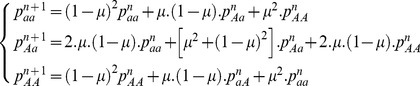
(1)
The genotypic frequencies inherited from sexual events 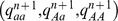 at the generation 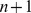 were expressed as a function of the mutation rate *μ* and the previous genotypic frequencies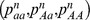.
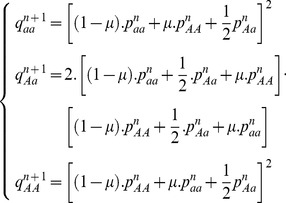
(2)Considering the rate of asexuality *c*, the genotypic frequencies at the next generation, 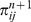, are
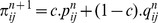
(3)where *i*,*j* can be alleles *A* or *a*.

Assuming the transition probability from one genotypic state 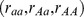 at generation *n* to another state 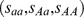 at the next generation *n+1* is given through the multinomial distribution 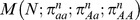, then
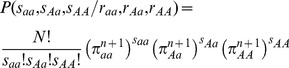
(4)The probability of a genotypic state in the next generation is therefore obtained through generations as
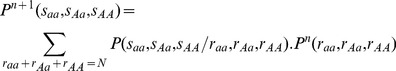
(5)where 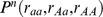 is the probability of the state 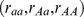 at the generation *n*.

The 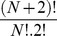 elements 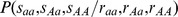 of the transition matrix are positive. The generated Markov chain is irreducible and aperiodic thus ergodic. As stated by the Perron-Frobenius theorem [34], the system is driven through generations to the stationary distribution of genotypic repartitions by the dominant eigenvector corresponding to the largest eigenvalue 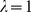 of the transition matrix [35]. This model also allows exploring the probability of genotypic repartitions dynamically through generations by computing the successive powers of the transition matrix, but for better clarity of the messages, we only focused on the consequences of partial asexuality on distribution of genetic diversity at equilibrium in this paper.

The probability density of genotypic repartitions contains all the information that population geneticists analyze and thus provides more information than any synthetic population genetics parameter would ever do. But, in this paper, considering the strong interest of the scientific community and the previously formulated theoretical expectations concerning the effects of partial asexuality on *F*_IS_ values [16], we focused on the full exact distribution of *F*_IS_ at equilibrium after an infinite number of generations. Thus, for each genotypic repartition of the individuals within a population, we computed allelic identities: *Q*_w_ which is the probability that two homologous alleles are identical within a diploid individual, and *Q*_b_ which is the probability that two homologous alleles are identical as they came from different individuals [36]. Then, the inbreeding coefficient *F*_IS_ was thus classically obtained from *Q*_w_ and *Q*_b_. 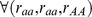:
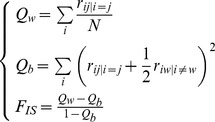
(6)We analyzed and discussed afterward the discrete probabilities density distributions of *Q*_w_, *Q*_b_ and *F*_IS_ obtained at equilibrium. We then computed the probability of negative *F*_IS_ as the sum of the probabilities of genotypic repartitions that resulted in strictly negative *F*_IS_. We considered the probability of positive *F*_IS_ as the sum of the probabilities of genotypic repartitions that resulted in zero and positive *F*_IS_ but excluding the two genotypic repartitions where one of the alleles was fixed within the population (the states of fixation). To understand how the rates of asexuality impacted the evolution of homozygote and heterozygote excesses in one generation at one locus, we computed the mean probabilities to change and keep the sign of *F*_IS_ as the sums of transition probabilities divided by their number knowing the signs of *F*_IS_ of the previous 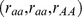 and next 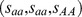 genotypic states.

All mathematical developments were computed using Python 2.7 (Python Software Foundation 2001–2012) and Numpy 1.7.0 (NumPy Developers, 2005–2012). The binary package (PASEX 1.0) of the algorithms we used to compute the transition matrix and the stationary distributions of genotypic repartitions is available at https://w3.rennes.inra.fr/IGEPP/PASEX/PASEX_1.0.zip.

In this paper, we chose to analyze results considering two population sizes of 60 and 140 individuals because they were equivalent to the population sizes encountered in some partially asexual populations of wild cherry trees used to assess the origin of the negative *F*_IS_ values commonly found in this species [17].With current workstation (HP Z800 Intel Xeon X5650 @2.67Ghz with 96 Go RAM), we were able to predict the probability of the genotypic distribution for 385 individuals overnight avoiding swapping. Raw transition matrix for 140, 250 and 400 individuals respectively required 1.74 Go, 17 Go and 113 Go to be stored and analyzed.

### Moments of the distributions of F_IS_

To compare our results with previous ones formulated using the two first moments of the possible distributions of *F*_IS_ [19], we also resumed the exact theoretical distributions we obtained by their four first moments. We thus computed the mean and the variance of *F*_IS_. To seek for more subtle effects of partial asexuality on *F*_IS_, we also studied the third and the fourth moments of the possible distributions of *F*_IS_ by computing the classical skewness 
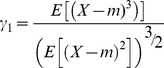
 and the classical excess of kurtosis 
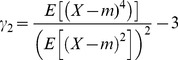
 where 3 stands for the kurtosis of a Gaussian distribution. In those equations, *m* is the mean value of the distribution of the variable *X*.

## Results

**Figure 1:**
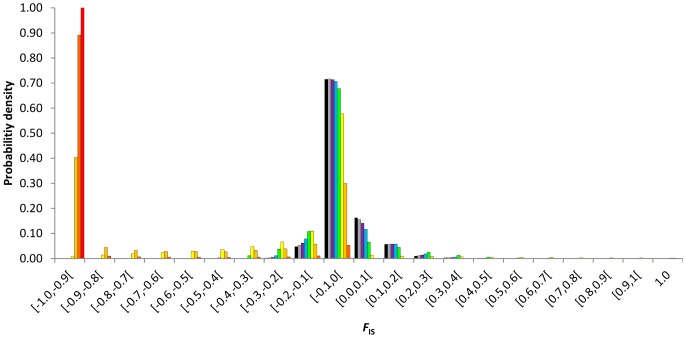
Discrete probability density distributions of *F*_IS_ at equilibrium as a function of the rate of asexuality, *c* = 0 (black), 0.3 (grey), 0.5 (purple), 0.7 (bleu), 0.9 (green), 0.99 (yellow), 0.999 (light orange), 0.9999 (dark orange), 1 (red). Results obtained for a population size of *N* = 140 and a mutation rate of *μ* = 10^−8^.

**Figure 2:**
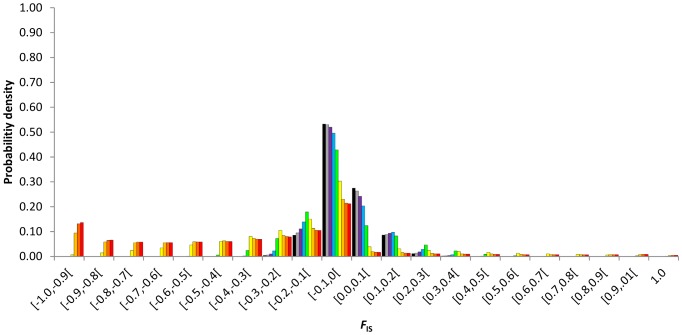
Discrete probability density distributions of *F*_IS_ at equilibrium as a function of the rate of asexuality, *c* = 0 (black), 0.3 (grey), 0.5 (purple), 0.7 (bleu), 0.9 (green), 0.99 (yellow), 0.999 (light orange), 0.9999 (dark orange), 1 (red). Results obtained for *N* = 140 and *μ* = 10^−3^.

**Figure 3:**
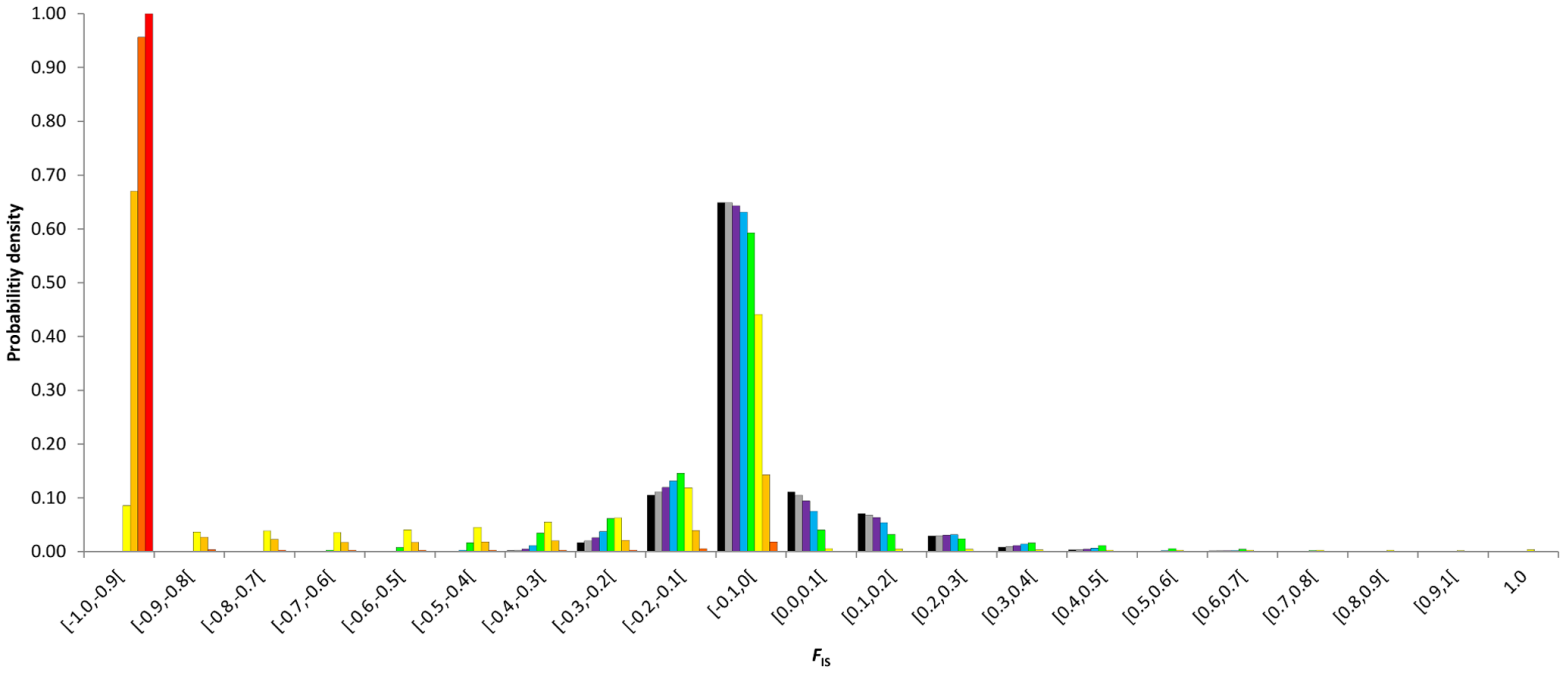
Discrete probability density distributions of *F*_IS_ at equilibrium as a function of the rate of asexuality, *c* = 0 (black), 0.3 (grey), 0.5 (purple), 0.7 (bleu), 0.9 (green), 0.99 (yellow), 0.999 (light orange), 0.9999 (dark orange), 1 (red). Results obtained for *N* = 60 and *μ* = 10^−8^.

**Figure 4:**
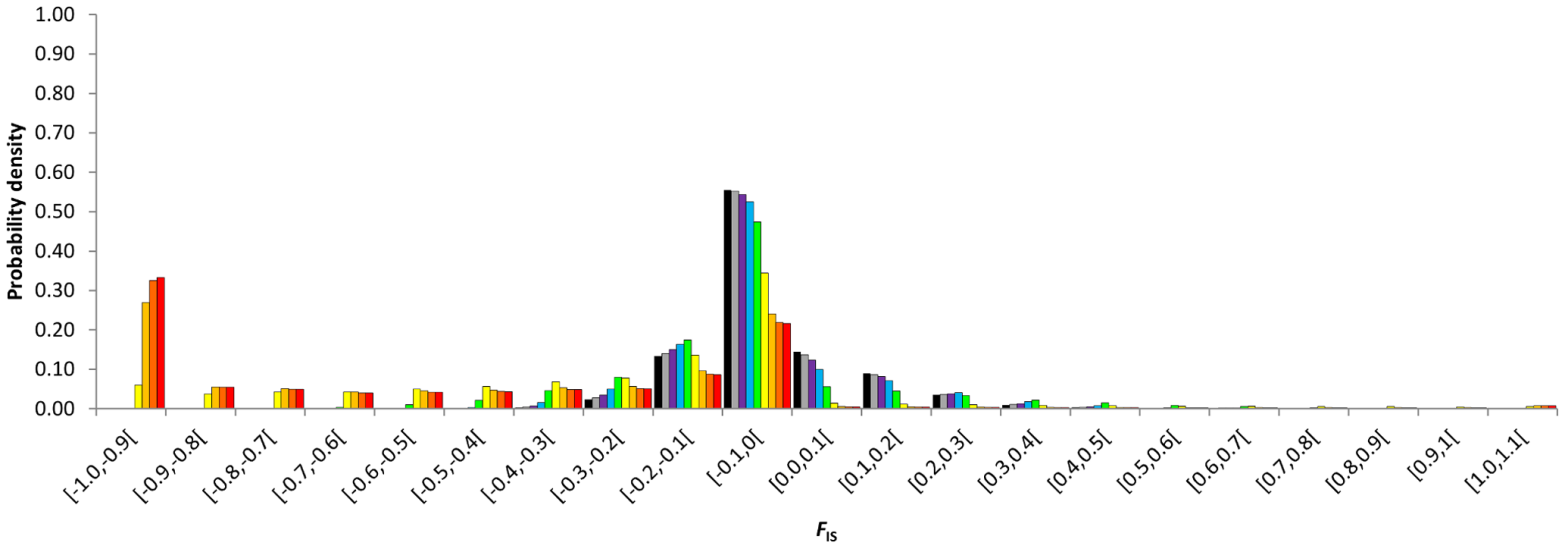
Discrete probability density distributions of *F*_IS_ at equilibrium as a function of the rate of asexuality, *c* = 0 (black), 0.3 (grey), 0.5 (purple), 0.7 (bleu), 0.9 (green), 0.99 (yellow), 0.999 (light orange), 0.9999 (dark orange), 1 (red). Results obtained for *N* = 60 and *μ* = 10^−3^.

The discrete probability densities of *F*_IS_ at equilibrium were impacted by the rates of asexuality since low rates (Figures 1, 2, 3, 4). Compared to similar sexual population (same population size and mutation rate), partial asexuality mainly modified the shape and the range of the possible distributions of *F*_IS_. Increasing rates of asexuality tended to shift the distribution of *F*_IS_ into negative values and to spread the right tail of the distributions into positive values. Therefore, it increased the probability to observe negative *F*_IS_ and decreased the probability to observe positive *F*_IS_, and also explained why some isolated highly positive *F*_IS_ values are actually obtained.

Intermediate asexual populations (0<*c*≤0.9) showed specific distributions of *F*_IS_ that varied from those obtained in highly and fully asexual populations and were distinguishable from the distribution obtained from fully sexual population (Figures 1, 2, 3, 4). Increasing rates of asexuality substantially increased the probability of negative *F*_IS_. This effect was especially visible for negative *F*_IS_ values close to zero. Moreover, the distributions of *F*_IS_ from intermediate asexual populations showed a deficit of small positive values (0≤*F*_IS_≤0.1) in comparison with distribution from fully sexual population, and this deficit increased with the rates of asexuality until 0.9. For higher positive *F*_IS_ values (0.1<*F*_IS_≤1), the discrete distributions of *F*_IS_ showed complex variations when the rate of asexuality increased. Varying the rates of asexuality modified the transition probabilities and thus, the probabilities of genotypic states obtained at equilibrium. For example, in a population of 140 individuals mutating at a rate of 10^−6^, changing *c* from 0 to 0.5 modified the ranks of probabilities of 9882 genotypic states. Only 129 remained identical, meaning that the paths to walk through the transition matrix winded differently along the possible genotypic states according to the rates of asexuality. Globally, genotypic states with negative *F*_IS_ tended to increase their ranks to the most likely while genotypic states with positive *F*_IS_ tended to decrease their ranks when the rates of asexuality increased. For example, at equilibrium, the genotypic state (*aa*:116 *aA*:24 *AA*:0) resulting in a negative *F*_IS_ of −0.09375 was ranked as the 82^nd^ most probable state when population reproduced sexually (*c* = 0) while it was ranked 48^th^ when the rate of asexuality was 0.5. Conversely, the genotypic state (*aa*:124 *aA*:15 *AA*:1), resulting in a positive *F*_IS_ of 0.06061284, was ranked the 48^th^ most probable state in fully sexual population and only the 60^th^ when the rate of asexuality was 0.5.

Populations reproducing using high rates of asexuality (0.9<*c*<1) strongly shifted their discrete probability densities into negative *F*_IS_ values. But they also spread the tails of their distributions over high values of positive *F*_IS_. Actually, we expected more high positive *F*_IS_ values (0.4≤*F*_IS_<1) in highly asexual populations than in more sexual populations.

From those distributions, we first computed the probabilities of fixation (Table 1). Increasing the rates of asexuality decreased the probabilities of fixation for a fixed population size and mutation rate. When genetic drift prevailed over mutation, the probabilities of fixation were drastically reduced only in highly asexual populations, mainly because asexuality creates as many states of fixation as a locus has possible genotypic states within an individual. In our case, with two alleles, each locus has thus three states of fixation under pure asexuality. When mutation prevailed over genetic drift, the probabilities of fixation decreased more smoothly as the rates of asexuality increased. Beyond fifty per cent of asexuality, the probabilities of fixation tended to decrease faster as the rates of asexuality increased.

**Table 1:** Probabilities of negative and positive *F*_IS_ excluding the states of fixation, and probabilities of allele fixation expected considering fixed population sizes *N*, mutation rates *μ*, and rates of asexuality *c* after an infinite number of generations.

*Probabilities of Negative F_IS_*							
*N*		60			140		
*μ*		10^−8^	10^−6^	10^−3^	10^−8^	10^−6^	10^−3^
	0.0	0.773781	0.773717	0.714740	0.764626	0.764442	0.622993
	0.3	0.782444	0.782382	0.724187	0.771232	0.771050	0.630751
	0.5	0.793511	0.793449	0.736088	0.780496	0.780318	0.641124
*c*	0.7	0.813326	0.813266	0.757360	0.797564	0.797392	0.660175
	0.9	0.862251	0.862196	0.810121	0.840439	0.840284	0.709600
	0.99	0.961651	0.961606	0.915361	0.935708	0.935592	0.824686
	0.999	0.998047	0.998024	0.957019	0.992977	0.992896	0.887028
	0.9999	0.999973	0.999969	0.963072	0.999858	0.999839	0.898220
	1.0	1.000000	1.000000	0.963778	1.000000	0.999999	0.899581

*Probabilities of Positive F*_IS_							
*N*		60			140		
*μ*		10^−8^	10^−6^	10^−3^	10^−8^	10^−6^	10^−3^
	0.0	0.226219	0.226283	0.285260	0.235374	0.235558	0.377007
	0.3	0.217556	0.217618	0.275813	0.228768	0.228950	0.369249
	0.5	0.206489	0.206551	0.263912	0.219504	0.219682	0.358876
*c*	0.7	0.186674	0.186734	0.242640	0.202436	0.202608	0.339825
	0.9	0.137749	0.137804	0.189879	0.159561	0.159716	0.290400
	0.99	0.038349	0.038394	0.084639	0.064292	0.064408	0.175314
	0.999	0.001953	0.001976	0.042981	0.007023	0.007104	0.112972
	0.9999	0.000027	0.000031	0.036928	0.000142	0.000161	0.101780
	1.0	0.000000	0.000000	0.036222	0.000000	0.000001	0.100419

*Probabilities of Fixation*							
*N*		60			140		
*μ*		10^−8^	10^−6^	10^−3^	10^−8^	10^−6^	10^−3^
	0.0	0.999987	0.998692	0.301475	0.999964	0.996475	0.047616
	0.3	0.999987	0.998688	0.300491	0.999964	0.996471	0.047455
	0.5	0.999987	0.998684	0.299384	0.999964	0.996465	0.047276
*c*	0.7	0.999987	0.998674	0.297247	0.999964	0.996454	0.046938
	0.9	0.999986	0.998636	0.289634	0.999963	0.996409	0.045799
	0.99	0.999982	0.998250	0.244446	0.999958	0.995992	0.040003
	0.999	0.999946	0.994668	0.187527	0.999922	0.992382	0.032571
	0.9999	0.999587	0.961656	0.173858	0.999562	0.959452	0.030656
	1.0	0.499994	0.499430	0.172119	0.499984	0.498432	0.030408

Then, we computed the negative *F*_IS_ and positive *F*_IS_ (Table 1). Globally, for fixed population size and mutation rate, increasing rates of asexuality increased the probability of negative *F*_IS_ and decreased the probability to observe positive *F*_IS_. In detail, both probabilities strongly depended on the population sizes and the mutation rates. Dynamically, under intermediate rates of asexuality, the mean probability to change in one generation the sign of *F*_IS_ quickly decreased with increasing rates of asexuality (Table 2). At high rates of asexuality, this probability still decreased with increasing rates of asexuality but remained at the same scale as populations reproducing using fifty per cent of asexuality. Whatever, in fully asexual populations, the mean probability to change the sign of *F*_IS_ in one generation still remained around 5%.

**Table 2:** Average probabilities to change the sign of *F*_IS_ and to keep it one generation further considering fixed population sizes *N*, mutation rates *μ*, and rates of asexuality *c* at a biallelic locus.

		*μ* = 10^−8^				*μ* = 10^−3^			
*N*	*c*	Stay *F_IS_*<0	Change to *F_IS_*<0	Change to *F_IS_*≥0	Stay *F_IS_*≥0	Stay *F_IS_*<0	Change to *F_IS_*<0	Change to *F_IS_*≥0	Stay *F_IS_*≥0
	0	0.199036	0.156860	0.363895	0.280209	0.199050	0.156847	0.363864	0.280239
	0.1	0.232245	0.123651	0.266858	0.377246	0.232122	0.123774	0.267294	0.376809
	0.2	0.259695	0.096201	0.189838	0.454266	0.259493	0.096403	0.190475	0.453628
	0.3	0.280082	0.075814	0.138402	0.505701	0.279856	0.076040	0.139027	0.505076
60	0.5	0.305006	0.050890	0.085475	0.558629	0.304796	0.051100	0.085962	0.558141
	0.7	0.318537	0.037360	0.060775	0.583329	0.318353	0.037543	0.061194	0.582910
	0.9	0.326790	0.029107	0.046520	0.597584	0.326627	0.029270	0.046913	0.597190
	0.999	0.329770	0.026126	0.041445	0.602658	0.329615	0.026281	0.041836	0.602267
	1	0.329798	0.026099	0.041399	0.602705	0.329643	0.026254	0.041790	0.602314
	0	0.182093	0.160231	0.353721	0.303955	0.182092	0.160231	0.353636	0.304040
	0.1	0.229637	0.112687	0.212664	0.445013	0.229454	0.112869	0.213200	0.444476
	0.2	0.263149	0.079175	0.128427	0.529250	0.262910	0.079414	0.128986	0.528690
	0.3	0.283647	0.058677	0.087625	0.570052	0.283419	0.058905	0.088056	0.569620
140	0.5	0.304871	0.037453	0.052847	0.604829	0.304688	0.037635	0.053141	0.604536
	0.7	0.315231	0.027092	0.037583	0.620093	0.315082	0.027241	0.037821	0.619855
	0.9	0.321255	0.021068	0.028991	0.628686	0.321129	0.021194	0.029204	0.628473
	0.999	0.323380	0.018944	0.025985	0.631692	0.323262	0.019061	0.026192	0.631485
	1	0.323399	0.018924	0.025957	0.631719	0.323282	0.019042	0.026164	0.631512

### Effects mutation and drift on the distributions of F_IS_

Population sizes, mutation rates and rates of asexuality interacted to shape the discrete probability density distributions of *F*_IS_ after an infinite number of generations (Figures 1, 2, 3, 4). It created complex variations within the possible distributions of *F*_IS_. First, for a fixed mutation rate whatever the rates of asexuality, increasing genetic drift by decreasing population size increased probabilities of fixations. It also spread the distributions of *F*_IS_ toward extreme values and increased the probabilities of *F*_IS_ on the edge of the distributions while it decreased the probabilities of middle values that surrounded *F*_IS_ = 0. In addition, decreasing population size contributed to shift more obviously the whole probability densities towards one direction that depended on the rates of asexuality: under 0.9 of asexual events, the probability densities shifted to positive values while population size decreased, whereas above 0.9 of asexual events, the probability densities shifted to negative values as the genetic drift tended to fix heterozygotes in asexual lines. Decreasing population sizes moved the threshold rate of asexuality that demarcated intermediate and high asexuality as identified previously by their respective typical distributions of *F*_IS_. Our conjecture was that this threshold seemed to occur when 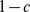 was roughly about 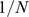. Dynamically, decreasing population size increased the probability to change the sign of *F*_IS_ in one generation (Table 2).

For a fixed population size, decreasing mutation rates massed the probability densities on *F*_IS_ intervals that were more likely at higher mutation rates. Those most common genotypic states themselves depended on an interaction between mutation rate, rate of asexuality and drift pressure. As a result, by decreasing mutation, thus giving scope for drift, probability densities shifted either to positive *F*_IS_ values for rates of asexuality lower than 0.9 (for population sizes of 60 and 140) or to negative *F*_IS_ values when rates of asexuality were above 0.9 (Table 1). Dynamically, increasing the mutation rate increased the probability to change the sign of *F*_IS_ in one generation (Table 2).

### Distribution summarized as the four first moments of the distributions

Because previous authors mainly focused on mean and variance of *F*_IS_ [19], [22], we wanted to assess if summering the distributions using their first moments could be trusted to describe the variations of distributions of *F*_IS_ obtained under various levels of asexuality, drift and mutation forces, and to compare them to previous theoretical results.

**Figure 5:**
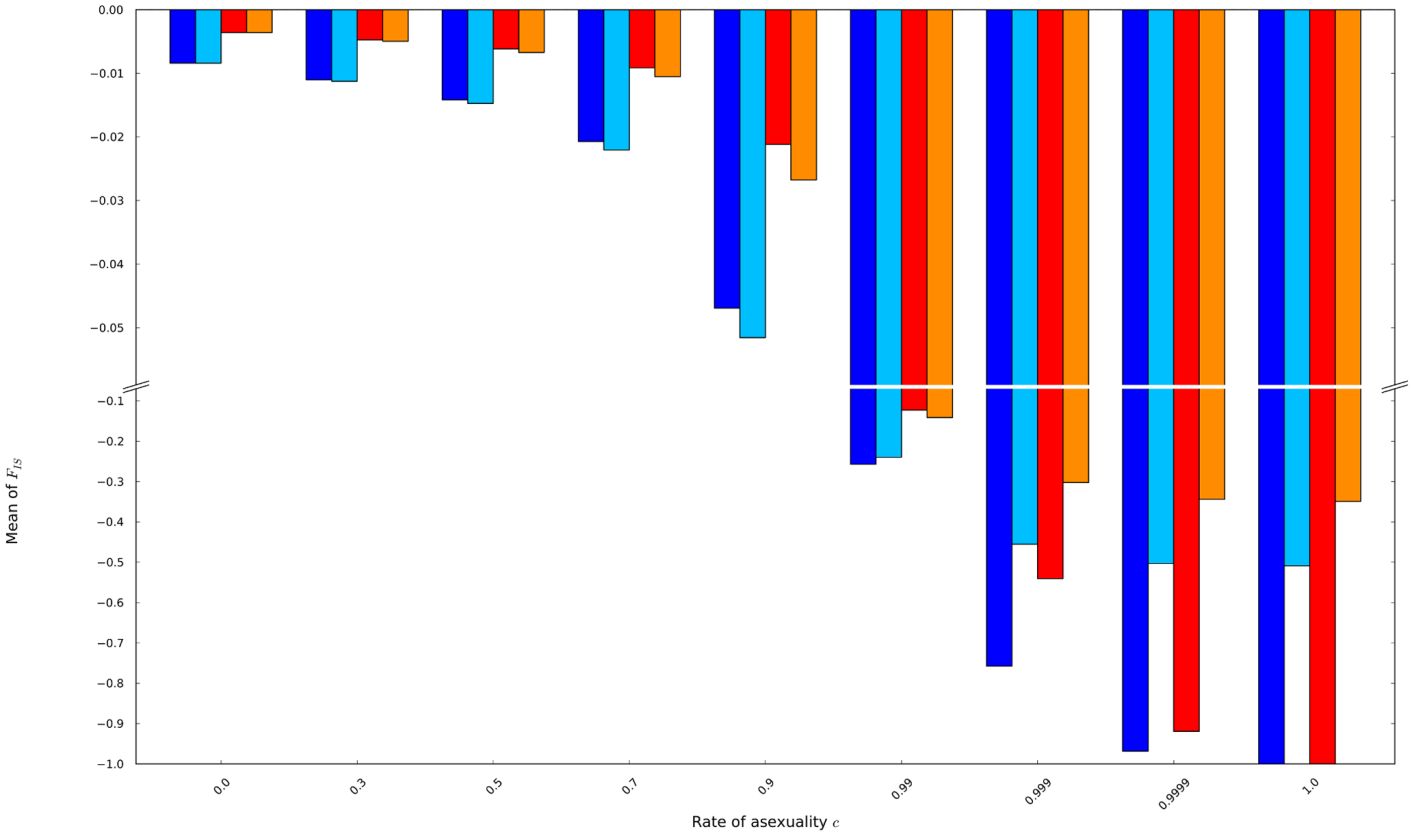
The means of the possible distributions of *F*_IS_ were obtained from populations of 60 individuals mutating at a rate of 10^−8^ (dark blue) and 10^−3^ (light blue), and from populations of 140 individuals mutating at a rate of 10^−8^ (red) and 10^−3^ (orange).

First, for population sizes of 60 and 140 individuals, the means of the possible distributions *of F*_IS_ decreased with increasing rates of asexuality whatever the population size and the mutation rate (Figure 5). Means of *F*_IS_ gave evidence for two strengths of effects of asexuality on genetic diversity: intermediate rates of asexuality (0<c≤0.9) smoothly impacted genetic diversity while high rates of asexuality (0.9<c≤1) did it roughly.

**Figure 6:**
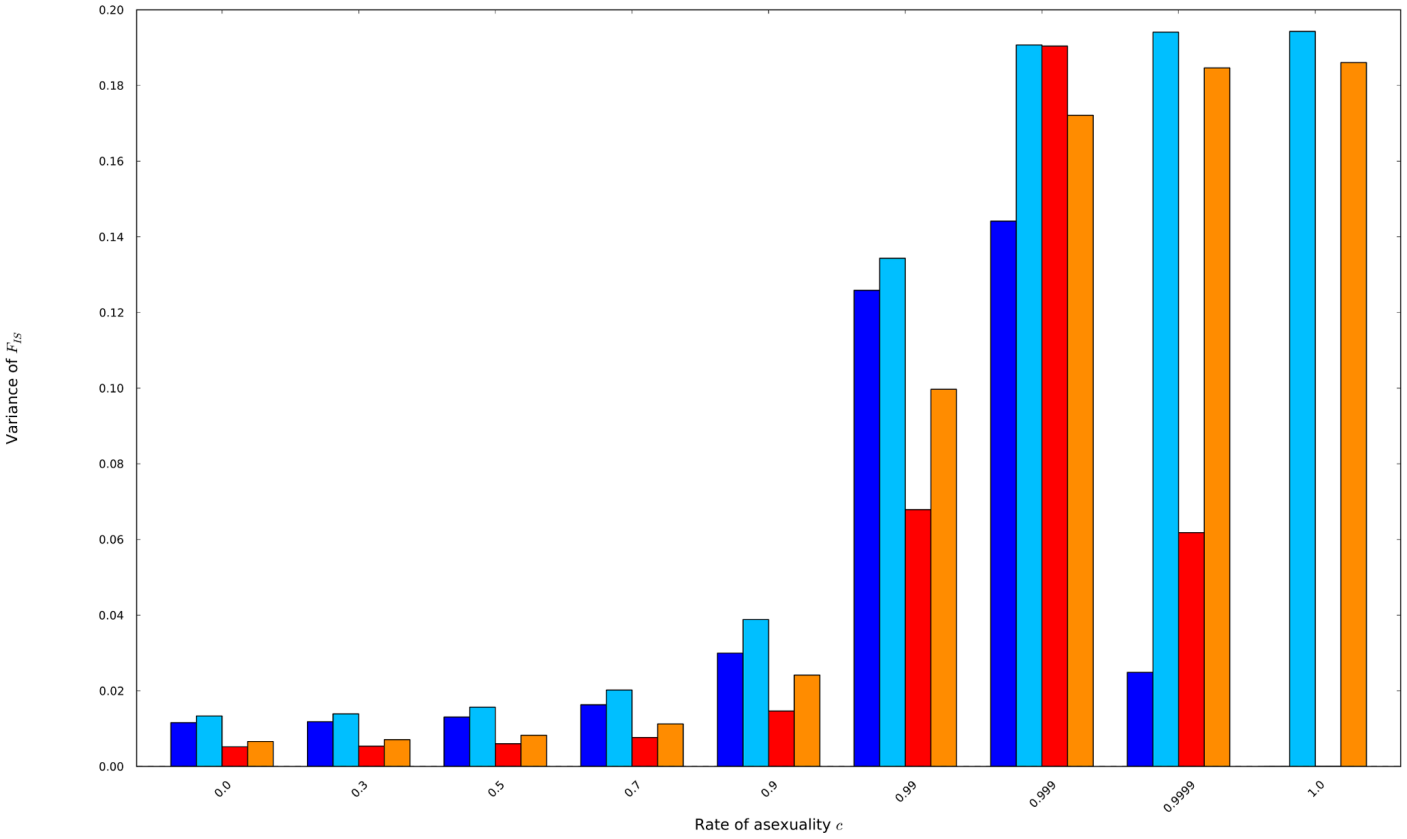
the variances of the possible distributions of *F*_IS_ were obtained from populations of 60 individuals mutating at a rate of 10^−8^ (dark blue) and 10^−3^ (light blue), and from populations of 140 individuals mutating at a rate of 10^−8^ (red) and 10^−3^ (orange).

Second, variance of *F*_IS_ globally tended to increase with the rates of asexuality (Figure 6). Interestingly, at high mutation rates (μ≥10^−5^ for *N* = 140), the variance of *F*_IS_ continuously increased with the rates of asexuality, reaching its highest values in fully asexual populations. However, lower mutation rates decreased the variance of the possible distribution of *F*_IS_ in nearly-fully and fully asexual populations because, out of fixation, the distributions of *F*_IS_ massed on *F*_IS_ = −1 mainly due to drift that fixed heterozygotes. Those distributions of *F*_IS_ massed on *F*_IS_ = −1 at low mutation rates, the states of fixation aside, tended to spread out as the population size increased. When population size decreased, the variance of *F*_IS_ tended to increase excepting when rates of asexuality were about 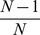.

**Figure 7:**
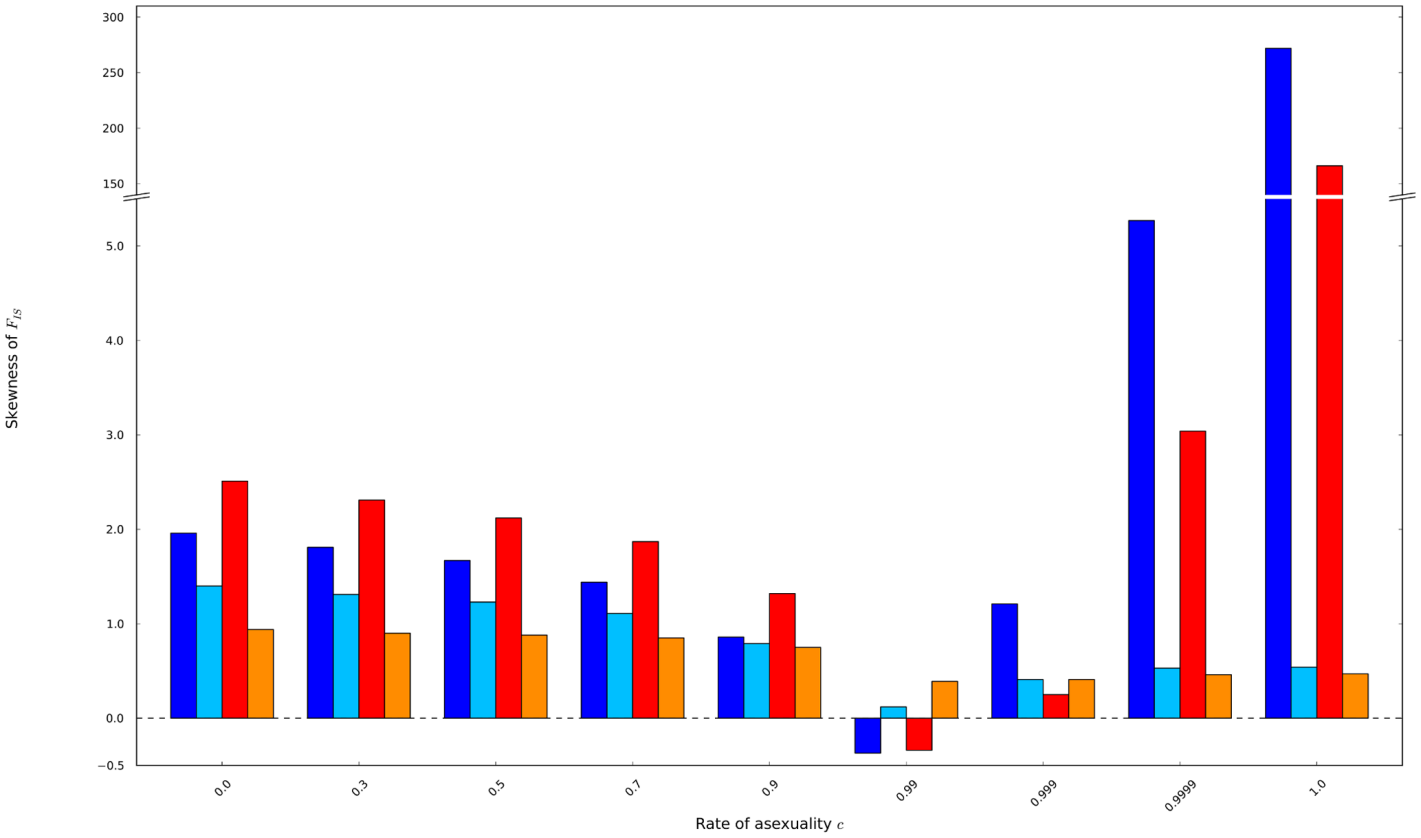
The skewnesses of the possible distributions of *F*_IS_ were obtained from populations of 60 individuals mutating at a rate of 10^−8^ (dark blue) and 10^−3^ (light blue), and from populations of 140 individuals mutating at a rate of 10^−8^ (red) and 10^−3^ (orange).

Third, the distributions of *F*_IS_ showed positive skewness meaning that the distributions massed on negative values and showed longer right tail to positive *F*_IS_ values (Figure 7). It decreased with increasing rates of asexuality until 0.9, demonstrating that the distributions tended to be symmetrically shaped around their mean. Then, when the rates of asexuality reached 1, the distribution of *F*_IS_ strongly skewed on *F*_IS_ = −1 and thus were only right-tailed, showing high positive values of skewness. This occurred when low mutation rates allowed the genetic drift to fix the heterozygotes. Conversely, when mutation rates prevailed over drift, the distribution remained quite symmetrical even under high and full asexuality.

**Figure 8:**
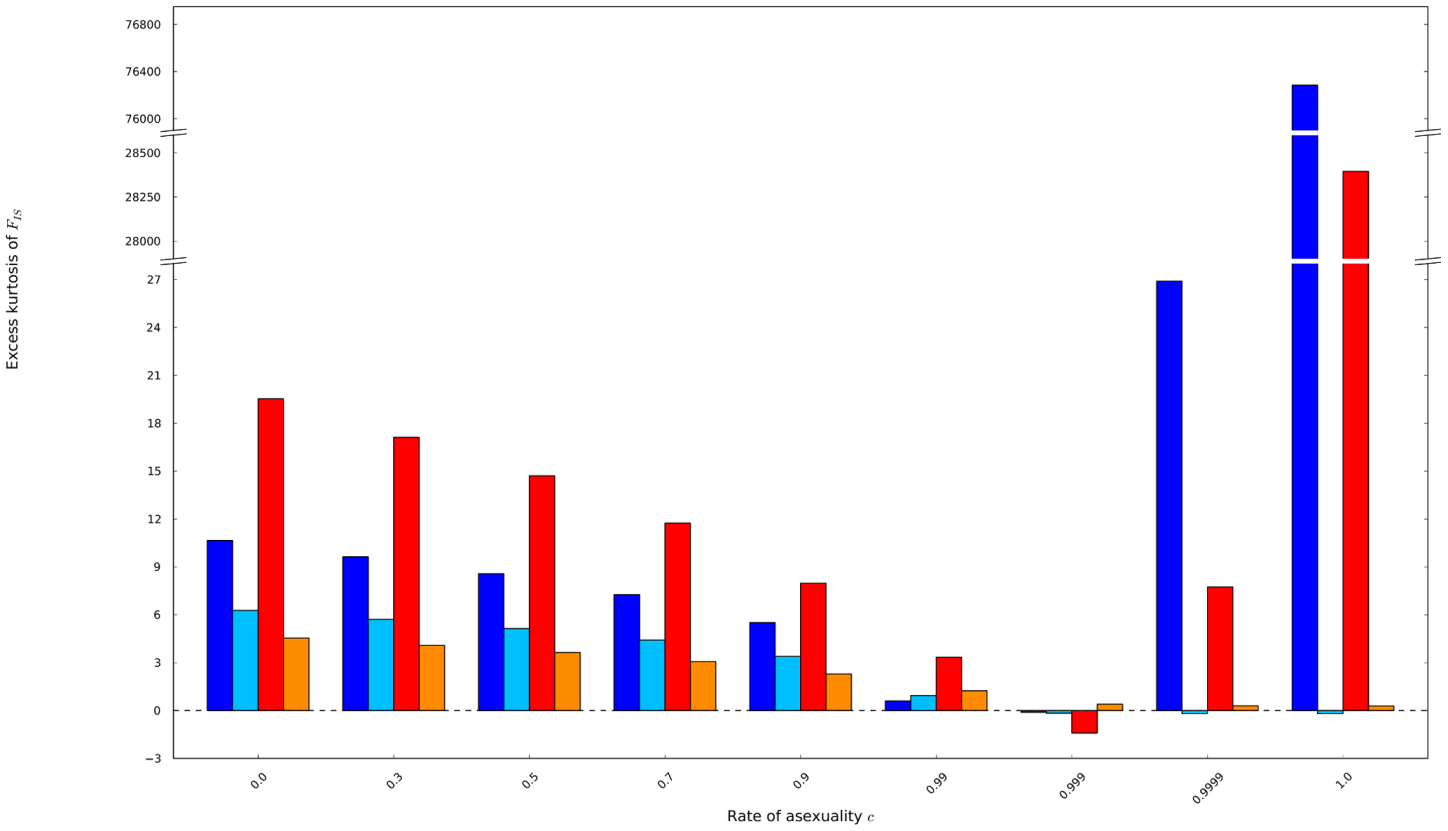
the kurtosis excesses of the possible distributions of *F*_IS_ were obtained from populations of 60 individuals mutating at a rate of 10^−8^ (dark blue) and 10^−3^ (light blue), and from populations of 140 individuals mutating at a rate of 10^−8^ (red) and 10^−3^ (orange).

Fourth, the distributions of *F*_IS_ showed positive excesses of kurtosis (compared to a similar Gaussian distribution) whatever the rates of asexuality meaning that the distributions showed fatter tails than expected from a Gaussian distribution (Figure 8). Interestingly, the mutation rates strongly influenced the shape of the distributions when populations reproduced through high or even full asexuality. Low mutation rates resulted in extremely peaky distributions of *F*_IS_ in full asexual populations as the values massed on strong negative *F*_IS_ and in fatter tails than expected considering such peak of values around the means. Those peaky and fat-tailed shapes smoothed with increasing population sizes or at higher mutation rates as the drift released its pressure to fix heterozygosity in the populations. Overall, when the rates of asexuality increased, the probability to observe negative *F*_IS_ increased faster than the decrease of the means of their distributions, acknowledging that the mean value of *F*_IS_ is quite inadequate to report clear expectations about the kind of *F*_IS_ values we should expect within data.

### Variation of allelic identities in partially asexual populations at equilibrium

**Figure 9:**
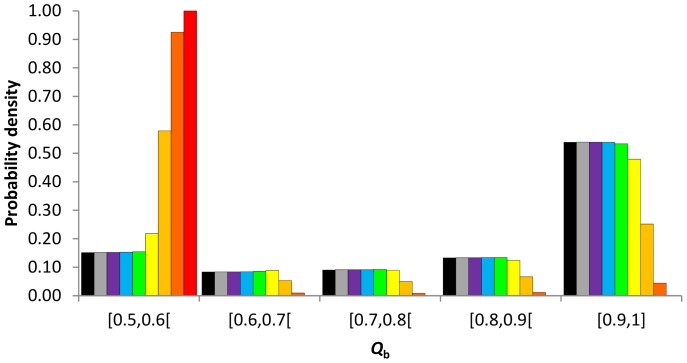
Discrete probability density distributions of allelic identities between individuals (*Q*_b_) at equilibrium for a population size of 140 individuals and a mutation rate of 10^−8^ as a function of the rate of asexuality, *c* = 0 (black), 0.3 (grey), 0.5 (purple), 0.7 (bleu), 0.9 (green), 0.99 (yellow), 0.999 (light orange), 0.9999 (dark orange), 1 (red).

At equilibrium, the discrete distributions of allelic identities between individuals (*Q*_b_) of populations reproducing with less than 0.9 of asexuality were very similar to those obtained in fully sexual population (Figure 9). Conversely, beyond 0.9 of asexuality, the distributions of *Q*_b_ strongly differed from those obtained under full sexuality. Beyond 0.9 of asexuality, increasing rates of asexuality increased the excess of low identities (*Q*_b_<0.7) while it decreased the probability of high values (*Q*_b_>0.7). Those distribution properties remained similar when the mutation rate increased even if at *μ* = 10^−3^ (Figure S1) the distributions of *Q*_b_ obtained in highly and pure asexual populations shaped more like the ones from intermediate and pure sexual populations.

**Figure 10:**
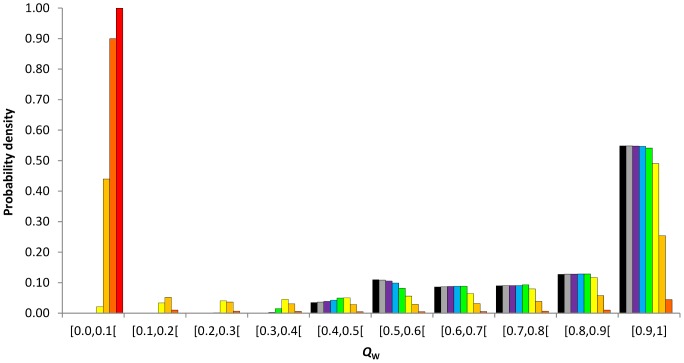
Discrete probability density distributions of allelic identities within individuals (*Q*_w_) at equilibrium for a population size of 140 individuals and a mutation rate of 10^−8^ as a function of the rate of asexuality, *c* = 0 (black), 0.3 (grey), 0.5 (purple), 0.7 (bleu), 0.9 (green), 0.99 (yellow), 0.999 (light orange), 0.9999 (dark orange), 1 (red).

The discrete distributions of allelic identities within individuals (*Q*_w_) showed more intricate patterns than the distributions of *Q*_b_ and its shape varied with the rate of asexuality (Figure 10). First, all distributions of *Q*_w_ came with two modes: a mode at high allelic identities (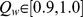) mainly due to the prevalence of the drift at such small population size (*N* = 140) over the mutation rates (*μ* = 10^−8^ and 10^−3^) and a second mode found at intermediate *Q*_w_ values (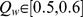). When the rate of asexuality increased, the second mode shifted to lower *Q*_w_ values while the lower tail of the distributions of *Q*_w_ fattened. As a result, intermediate asexual populations (0<*c*≤0.9) compared to fully sexual population globally showed a lower peak at the second mode (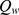
∼0.55), lower probabilities of *Q*_w_ superior to the second mode and higher probabilities of *Q*_w_ inferior to the second mode. Interestingly, the proportion of *Q*_w_ values around the smaller mode faithfully varied according to the rates of asexuality, even regarding low rates, and this property remained even at *μ* = 10^−3^ (Figure S2).

## Discussion

Our model allowed exploring dynamically and at equilibrium the full exact discrete distribution of *F*_IS_ obtained from finite population sizes at varying mutation rates and rates of asexuality at biallelic loci. It can be easily extended to loci made of multiple alleles but its computation complexity increases with 
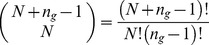
 where 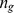 is the number of possible genotypes, itself resulting from 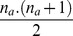 where 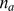 is the number of alleles. Whatever, our mathematical formalism constitutes the first and necessary step to derive a continuous diffusion process to assess the effects of partial asexuality on genetic diversity in larger finite populations [32], [37], [38].

After an infinite number of generations, partial asexuality even at low rate modified the distribution of *F*_IS_ values obtained from similar fully sexual populations. Globally, partial asexuality increased the probability to observe negative *F*_IS_ while it decreased the probability to observe positive *F*_IS_ and the probabilities of fixation. As already demonstrated in previous theoretical [39] and empirical studies [40], reproduction through asexuality increased the allelic diversity expected within populations, as reported by the *F*_IS_ index, when compared to similar fully sexual populations, because asexuality sets heritability and genetic drift at a genotypic level and conserves ancestral genetic states against drift, which finally decreases allelic identities within individuals. Our study also supports the fact that the rates of asexuality (or its opposite, the rate of genetic segregation in its broad definition) should be considered as a full evolutionary force because it distinctively impacts the transmission of genetic diversity through generations.

### Typology of the distributions of F_IS_

We distinguished three main types of distributions whose ranges varied with the rates of asexuality for fixed population sizes and mutation rates. The first type of distributions seemed to happen when 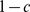 was smaller than 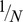. Previous authors conclude from predicted mean and simulated variance of *F*_IS_ that intermediate asexual populations should show the same pattern of genetic variation as found in fully sexual populations [19], [22], [24]. Excluding selective considerations, it implies that populations of individuals producing 90% of their descent asexually should decrease the putative costs of their sexual reproduction with low or even no genetic consequences. By the way, benefits of full sexuality should not be searched in its consequences on the evolution of genetic diversity [27]. We also found few differences between fully sexual and intermediate asexual populations considering only the mean of their distribution of *F*_IS_. But considering their whole discrete distribution of *F*_IS_, we found that intermediate asexual populations (0<c<0.99 for a populations of ∼100 individuals) showed excesses of faintly negative *F*_IS_ (−0.3≤*F*_IS_<−0.1) and deficits of slightly positive *F*_IS_ (0≤*F*_IS_<0.1) when compared to the corresponding fully sexual populations. Interestingly, at those rates of asexuality, most of the distributions of *F*_IS_ ranged between −0.3 and 0.1. By the way, the probabilities to observe negative *F*_IS_ increased against the probabilities to observe positive *F*_IS_. This effect can be illustrated by a simple example considering a population at (*aa*:0 *aA*:1 *AA*:*N*-1): through sexual reproduction, if we draw the heterozygous parent, we have only one chance on two to take its minor allele and thus to result in an heterozygous descent. Through an asexual event, we have the same probability to draw the heterozygous parent but a full chance that this draw actually results in a heterozygote. Thus asexuality retains to the next generation, by a (nearly) factor two, genotypes carrying minor allele compared to sexuality. Moreover, increasing asexuality until 0.9 slightly increased the variance of *F*_IS_, but clearly decreased its skewness and its kurtosis, which finally, increased the occasional occurrence of high positive *F*_IS_ values while most of the distributions moved slightly to negative *F*_IS_.

The second type of distributions happened when 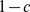 was about 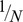. Such highly asexual populations (0.99≤c<1 for populations of ∼100 individuals) showed specific distributions of *F*_IS_ that strongly shifted to negative values when compared to similar fully sexual and intermediate asexual populations. They also spread the right tails of their distributions into high positive *F*_IS_ values (0.3<*F*_IS_≤1) when compared to similar fully sexual and intermediate asexual populations, even if most of their expected *F*_IS_ values laid between −1.0 and 0.

Finally, the third type of distributions happened when 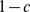 was greater than 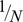. In this case, the distribution of *F*_IS_ tended to mass on *F*_IS_ = −1.0, arguing for populations only made of heterozygotes at their remaining polymorph loci. Besides, nearly half the genomic sites that have been polymorphic at one generation along the history of the species were expected to be heterozygous in all individuals after an infinite number of generations. However, the existence of this third type of distribution of *F*_IS_ required that genetic drift prevailed over mutation forces (low mutation rate and/or small population size) and thus strongly relied on the balance between mutation and drift forces within the population.

### Increasing rates of asexuality increased the variance of F_IS_

Previous simulations (20 multiallelic loci of 99 alleles in a metapopulation of 50 demes of 50 individuals, migration rate 0.1, mutation rate 10^−5^) performed using Easypop [41] show that the highest standard errors of *F*_IS_ should be found in highly asexual rather than in fully asexual populations. From those results, a framework was proposed to qualitatively infer the rate of asexuality from genetic data: four *scenarii* among the seven described rely on small or high variations of *F*_IS_ among genotyped loci to disentangle highly asexual populations from fully asexual ones [29]. Our mathematical model formalizing the full distributions of *F*_IS_ at biallelic loci in single finite populations showed that the third type of distribution (all the distribution massed on 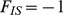) only occurred in small fully asexual populations at low rates of mutation (*e.g.* smaller than 10^−5^ for a hundred of fully asexual individuals). When genetic drift was low in front mutation, the distributions of *F*_IS_ in fully asexual populations were very close to those obtained from similar highly asexual populations and thus mainly spread from −1.0 to 0 with substantial occurrences of high *F*_IS_ values, covering all the spectrum of possible *F*_IS_ values. Interestingly, large populations of insects seem more prone to reproduce asexually than small ones [42], may be because the consequences of asexuality on the evolution of their genetic diversity are then so close to those obtained with more sexuality that the two-fold cost of sex would not be counter-balanced by any genetic advantage coming from some sexual reproduction?

When genetic drift was low in front mutation, the variance of *F*_IS_ continuously increased with increasing rates of asexuality and reached its highest value when populations were fully asexual. In our example, even with a physical occurrence of a mutation in the population every ∼10 generations, the maximal variance of *F*_IS_ was still obtained in fully asexual populations rather in highly asexual populations. Interestingly, in a highly asexual nematode species genotyped using microsatellites, the population with the highest linkage disequilibrium among the six studied, with the lowest number of genotypes not included in a clone and with the second lowest ratio of the number of multilocus genotypes found over the sample size also shows the highest variance over a mean significant negative *F*_IS_ (*population H* [8]). Conversely, in other populations, when measures apart from *F*_IS_ argued for less asexuality (their *c* were estimated around 0.9), their variances of *F*_IS_ were smaller than the one obtained in this nearly-fully asexual *H* population. Those observations, that were awkward to explain from previous theoretical predictions [19], [29] without resorting to complex biological reasons than just their reproductive mode, may be explained more parsimoniously by our theoretical results considering the fact that the mutation rates of microsatellites are supposed to mainly range between 0.1 and 10^−4^, seldom below 10^−6^ [43]–[45]. Multiallelic versus biallelic loci, metapopulation versus single population and variance estimated over ten simulations versus full exact distribution, respectively used in [19] and in our model, may also be sufficient to explain such discrepancy in variance. Consequently, we recommend considering carefully the balance between mutation and genetic drift, population structure and the number of alleles per loci when we want to disentangle highly from fully asexual populations.

### Some homozygote excesses still expected in fully asexual species

Our model predicted that increasing rates of asexuality should decrease the probability to observe positive *F*_IS_. But even in fully asexual populations when population sizes or mutation rates were high, our model showed that substantial probabilities to observe positive *F*_IS_ were still expected out of some allele fixation. Indeed, using the inductive properties of our model and linearizing the system (equation 3), we obtained:
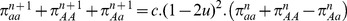
(7)Considering an infinite asexual population size that mutates, a convergence point can be reached only if 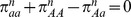 that implies that 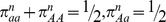. Thus, as soon as mutation occurs, full asexuality should result in 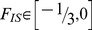 in very large populations. Only a preponderant genetic drift may drive the population to the unstable point 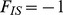 as most events of mutation, rearrangement and recombination such as gene conversion as observed in the bdelloid rotifer *Adineta vaga* [46] have a substantial probability to provide homozygotes and get back the population to 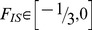. Notice that a preponderant genetic drift through multinomial drawings should equally drive the full asexual population to fix one of the two alleles and thus to dynamically walk through some genotypic states showing homozygote excesses. Those results are in accordance with the variance of *F*_IS_ that continuously increased with the rates of asexuality. As consequence, a small but substantial group of loci along the genomes should present homozygote excesses even in highly and fully asexual species. Likewise, at one locus at the scale of a species, a small number of isolated populations should present homozygote excesses while most of the other populations should present heterozygote excesses. This result offered new insights considering the transposition of the Meselson effect [11] at the scale of a population and the expected Muller's Ratchet accumulation of mutations over the asexual lines [47]. As soon as a clade evolves from multiple individuals with mutation, and not only from a single individual as specified in Meselson effect [11], we should not expect only heterozygotes in a population at most polymorphic sites along the genomes even after an infinite number of generations. Indeed, without selection at one locus, all incoming mutation in an asexual line has to struggle against the genotypic drift with the disadvantage that its original ancestral genotype is often already present at a high frequency in the population [48]. Besides, considering loci with a finite number of alleles like SNP, mutation has also substantial probabilities to provide homozygote from heterozygote over generations, especially if a strong mutational bias changes the DNA message towards the same nucleobase [49]. In the light of our results obtained after an infinite number of generations and the recent experimental advances on asexuality and hybridization, highly divergent alleles within genomes in asexual individuals may rather be understood as recent asexual lines triggered by interspecific hybridization as found for example in some vertebrate species [50] or as asexual lines evolving under diversifying selective forces, like heterosis or incoming from rare sexual events involving allogamy [8], than ancient asexual lines that would have accumulated independent mutation on their homologous copies of genomes. Even in a demonstrative study reporting only *F*_IS_ = −1 [51], the high genetic variability observed between separate clonal lineages, the evidences of heterozygote loci that switched to homozygosity due to mutation and the measures of strong negative *F*_IS_ even in recombining populations rather support such scenarios than ancient asexuality through few breeders.

Moreover, under an evolution without selection, our model demonstrated that we still expected in average 5% chance to change of *F*_IS_ sign from one generation to another, provided that some mutation (or gene conversion) may result in creating some homozygotes, so that some loci may present heterozygote excesses at one generation then homozygote excesses at the population scale. More than ever in partially, highly and fully asexual species, sampling effects have thus to be considered because a bias scheme or an insufficient numbers of populations, individuals and loci may result in erroneous biological conclusions. *Daphnia pulicaria* populations show huge variations of *F*_IS_ over the years [28]. For example, the Bristol population shows in 2002 the genotypic proportions expected under Hardy-Weinberg equilibrium, then strong heterozygote excess in 2004 and finally strong homozygote excess in 2005. They discuss that those variation of *F*_IS_ may be due either to varying frequencies of sex in their populations, or to some random fluctuations in selection intensity or to the fact that different combinations of genes may result in the same favored phenotype at different times. Similarly, in cyclically asexual populations, clonal erosion, recurrent reduction in population sizes or some selective events favoring the dominance of some asexual lineages were proposed as causes to explain the change of sign of *F*_IS_ through generations [23], [52]. However, the stable genotypic diversity ratio over years and the high number of pairs of loci significantly in linkage disequilibrium in *Daphnia pulicaria* populations support stable biological features over the years [28]. A parsimonious explanation would be that these populations reproduced using the same rates of asexuality summed over the year (same proportion of partially and fully asexual lines within), years after years, and only evolved showing the typical stochastic variations of *F*_IS_ we expect under partial asexuality considering mutation and drift forces alone.

### Perspectives to infer rates of asexuality from F_IS_ values

Our model provided new perspectives to infer rates of asexuality from genetic dataset, especially at low rates (0<*c*<0.9 for population of about one hundred individuals). Indeed, the inference of the effective rates of asexuality from genetic data without *a priori* remains one of the main expectations accounting for the theoretical development of population genetics tools [4], [16], [25]. If the mean value of *F*_IS_ alone should not allow inferring the rates of asexuality because of its lack of variation at low rates of asexuality [19], [29], full distributions of *F*_IS_ and analyses considering two generations (on the change of *F*_IS_ sign for example) are promising. Proportion of negative *F*_IS_ and of small positive *F*_IS_ values within a population seemed to vary faithfully with the rates of asexuality, even at intermediate rates of asexuality. With the increasing facilities to easily obtain molecular data from populations, field population genetics studies may gain to confront observed and theoretical distribution of *F*_IS_ along the genomes. However, our results also demonstrated that the theoretical predictions strongly depended on the balance between the mutation rate (and probably mutation models) and the genetic drift. Therefore, inference of the rate of asexuality should be considered assuming some known mutation rate and population size. Moreover, the changes in *F*_IS_ values over generations seemed the suitable genetic tool to detect few asexual events in mainly sexual populations and to indirectly distinguish them from full sexuality.

Some authors argue that indirect methods to infer rates of asexuality through population genetics models are not yet mature to be trusted [53], [54]. Indeed, because models are not taking into account for most evolutionary forces and genetic complexities yet, indirect inference of high and full asexuality can even provide false estimates considering selection, epistasis and bias or insufficient sample scheme. These authors thus call to rather rely on morphological data and even to conclude from the lack of observation of morphological male in samplings or when observed, considered as not sexually functional from an expert point of view, that if there are any sexual individuals in bdelloids, darwinulids and oribatids populations, they are very rare and thus can be considered as void of biological meaning [53]. Our results, as all previous population genetics models [19], [23], [24] and simulations [26], suggest that the corner stone of the genetic evolution of such species strongly depends on their effective rates of asexuality. A rare sexual event over some generations seems sufficient to deeply impact the genetic polymorphism and its structure within population, and so the evolutionary potential of such species.

## Conclusion

By using the full and exact distribution of *F*_IS_, we showed that low rates of asexuality modify genetic diversity. We put out three main patterns of effects of the rates of asexuality on intrapopulational genetic diversity at equilibrium. The occurrence of those patterns depended on the balance between mutation, genetic drift and rates of asexuality. Rare asexual events in a mainly sexual population changed the shape and the symmetry of the distributions of *F*_IS_. Moreover, when the rates of asexuality increased, the probabilities that one locus changes the sign of its *F*_IS_ value in one generation quickly decreased in mainly sexual populations. It supports that reproductive systems at intermediate rates of asexuality have specific evolution of their genetic diversity that differ from those of similar fully sexual populations.

Our model also showed that increasing the rates of asexuality increased the variance and deeply modified the shape and the symmetry of the distributions of *F*_IS_. In consequence, among mainly negative *F*_IS_, the substantial occurrence of some high positive *F*_IS_ values was still observed in highly and fully asexual populations. The specific effects of intermediate and high rates of asexuality and the full exact distributions of positive and negative values, assuming a rate of asexuality, a population size and a mutation rate, constitute new promising triggers to quantitatively infer the rates of asexuality from multiple *F*_IS_ measures from genomic data within populations. Interestingly, heterozygote excesses at most loci along the genomes of ancient fully asexual populations can only occur in small populations of few isolated individuals evolving at low mutation rates. Finally, our results indicate that the evolutionary consequences of biological trait would benefit from being studied using the full distributions of their impacts on population genetics parameters.

## Supporting Information

Figure S1 **Discrete distributions of the density probabilities of allelic identities between individuals (** ***Q*** **_b_) at equilibrium for a population size of 140 individuals and a mutation rate of 10^−3^ as a function of the rate of asexuality,** ***c*** **= 0 (black), 0.3 (grey), 0.5 (purple), 0.7 (bleu), 0.9 (green), 0.99 (yellow), 0.999 (light orange), 0.9999 (dark orange), 1 (red).**
(TIF)

Figure S2 **Discrete distributions of the density probabilities of allelic identities within individuals (** ***Q*** **_w_) at equilibrium for a population size of 140 individuals and a mutation rate of 10^−3^ as a function of the rate of asexuality,** ***c*** **= 0 (black), 0.3 (grey), 0.5 (purple), 0.7 (bleu), 0.9 (green), 0.99 (yellow), 0.999 (light orange), 0.9999 (dark orange), 1 (red).**
(TIF)
