# Supplementary figures and images for: The Exact Distributions of F IS under Partial Asexuality in Small Finite Populations with Mutation
Source: PLoS One. 2014 Jan 21;9(1):e85228. doi: 10.1371/journal.pone.0085228 (PMC3897417; doi:10.1371/journal.pone.0085228)

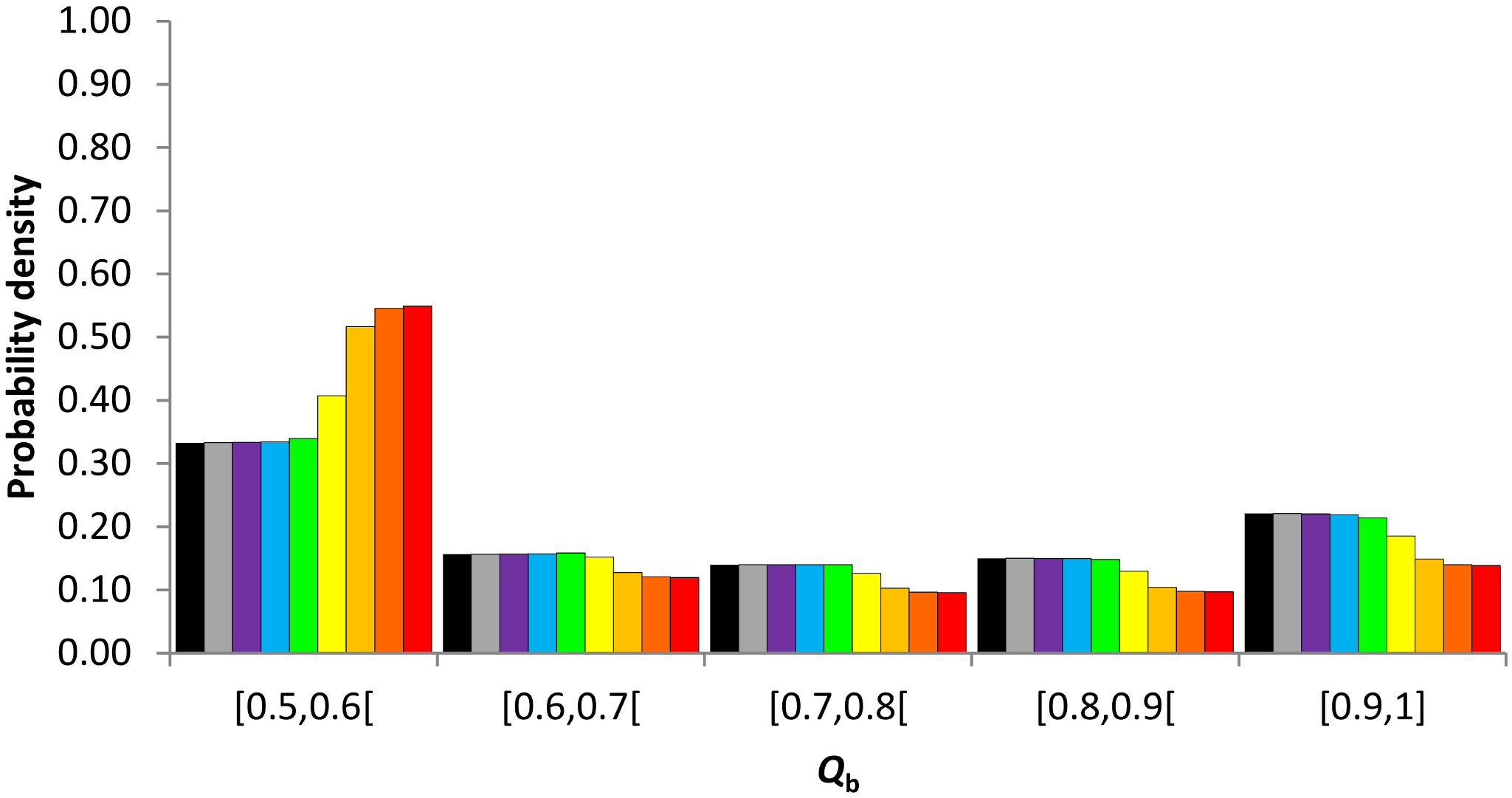

Supplement: Figure S1 — Discrete distributions of the density probabilities of allelic identities between individuals ( Q b) at equilibrium for a population size of 140 individuals and a mutation rate of 10−3 as a function of the rate of asexuality, c = 0 (black), 0.3 (grey), 0.5 (purple), 0.7 (bleu), 0.9 (green), 0.99 (yellow), 0.999 (light orange), 0.9999 (dark orange), 1 (red). (TIF) [file pone.0085228.s001.tif]

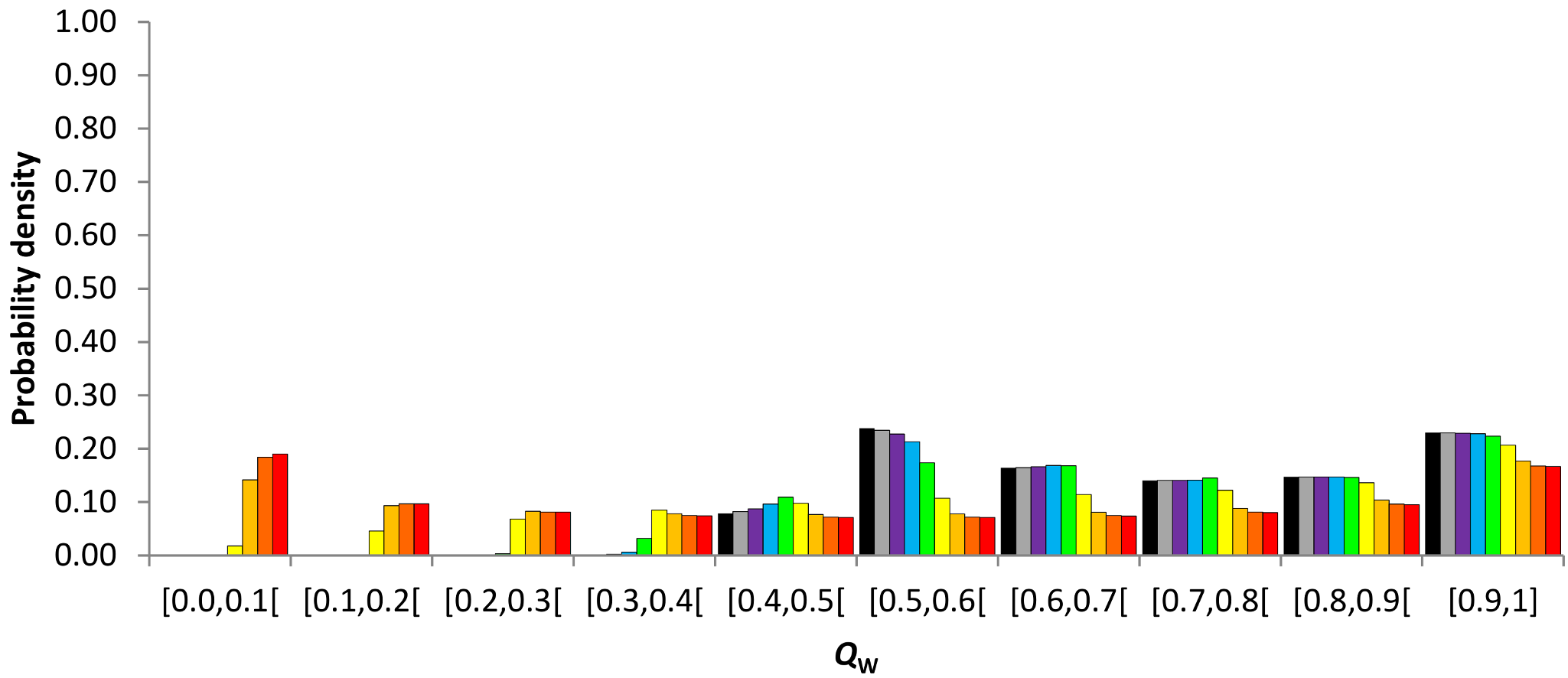

Supplement: Figure S2 — Discrete distributions of the density probabilities of allelic identities within individuals ( Q w) at equilibrium for a population size of 140 individuals and a mutation rate of 10−3 as a function of the rate of asexuality, c = 0 (black), 0.3 (grey), 0.5 (purple), 0.7 (bleu), 0.9 (green), 0.99 (yellow), 0.999 (light orange), 0.9999 (dark orange), 1 (red). (TIF) [file pone.0085228.s002.tif]
